# Causes of Death in Anti-IgLON5 Disease: A Novel Case Report and Systematic Literature Review

**DOI:** 10.3390/brainsci15090921

**Published:** 2025-08-26

**Authors:** Tina Howischer, Lukas Gattermeyer-Kell, Stephanie Hirschbichler, Thomas Seifert-Held, Jan Hinrich Bräsen, Petra Katschnig-Winter, Mariella Kögl, Sebastian Franthal, Christian Enzinger, Romana Höftberger, Petra Schwingenschuh

**Affiliations:** 1 Department of Neurology, Medical University of Graz, 8010 Graz, Austria; tina.howischer@stud.medunigraz.at (T.H.); lukas.gattermeyer@medunigraz.at (L.G.-K.); stephanie.hirschbichler@medunigraz.at (S.H.); petra.katschnig@medunigraz.at (P.K.-W.); mariella.koegl@medunigraz.at (M.K.); sebastian.franthal@medunigraz.at (S.F.); chris.enzinger@medunigraz.at (C.E.); 2 Karl Landsteiner University of Health Sciences, 3500 Krems, Austria; 3 Department of Neurology, University Hospital St. Pölten, 3100 St. Pölten, Austria; 4 Department of Neurology, Hospital Murtal, 8720 Knittelfeld, Austria; thomas.seifert-held@kages.at; 5 Department of Pathology, University of Hannover, 30625 Hannover, Germany; braesen.jan@mh-hannover.de; 6 Division of Neuropathology and Neurochemistry, Department of Neurology, Medical University of Vienna, 1097 Vienna, Austria; romana.hoeftberger@meduniwien.ac.at

**Keywords:** anti-IgLON5 disease, transthyretin amyloidosis, ATTR amyloidosis, causes of death, sudden death, case report, systematic review

## Abstract

Background/Objectives: Anti-IgLON5 disease is a neurological disorder characterized by the presence of autoantibodies directed against the neuronal cell adhesion protein IgLON5. Pathophysiology involves both autoimmune inflammation and neurodegenerative processes. The most common causes of death are sudden death, central hypoventilation, dysphagia, and aspiration. However, the high rate of largely unclear sudden deaths calls for further research in this area. Methods: We performed a systematic search of the literature on causes of death in anti-IgLON5 disease following the PRISMA guidelines. In addition, we present a new case that was followed up in our clinic until death. Results: Of 258 publications with anti-IgLON5 disease, 21 publications comprising 61 cases that reported the causes of death were included in the analysis. The most common cause of death was death due to complications at 36.1%, followed by sudden death, accounting for 32.8% of the cases. Other causes include respiratory, cardiac, and unknown causes. The patient presented here as a case report was also diagnosed with cardiac amyloidosis and died from a cardiac cause of sudden death. Conclusions: Sudden death in anti-IgLON5 disease is one of the most common causes of death in the literature. A progressive neurodegenerative process in the brain stem causing central hypoventilation is generally assumed as a major causative factor. The case reported here had concomitant cardiac amyloidosis, which may raise the question as to whether unrecognized cardiac causes, which are not routinely screened for in this population, might represent another cause of sudden death, which would have important therapeutic implications.

## 1. Introduction

Anti-IgLON5 disease was first described in 2014 [1], representing a disorder in which both autoimmune and neurodegenerative processes are involved. Antibodies are produced against the neuronal cell adhesion protein IgLON5, whose exact function remains unknown. An association between the disease and human leukocyte antigen (HLA)-DRB1*10:01 and HLA-DQB1*05:01 alleles has been described [2]. Sleep disorders often dominate the clinical picture as the disease progresses [1], including rapid eye movement (REM) sleep disorder and non-REM sleep disorders [3], as well as obstructive sleep apnea syndrome (OSAS) [1]. Other symptoms include bulbar symptoms, gait instability [2], supranuclear gaze palsy, and movement disorders [4]. Diagnosis relies on the detection of antibodies against the neuronal cell adhesion protein IgLON5, which can be found in both the cerebrospinal fluid (CSF) and serum [5]. Cerebral magnetic resonance imaging (MRI) may reveal atrophy of the brain stem and cerebellum but is often unremarkable [3]. Treatment with immunotherapy has mainly been reported to be either ineffective or associated with only partial improvement and prolonged disease stabilization. However, findings from a recent study suggest better outcomes if immunotherapy is started early in the disease course [6,7].

Reported mortality rates vary between 19% and 59%, with sudden death, central hypoventilation, dysphagia, and aspiration most frequently cited as causes of death [2,3,6]. The high incidence of sudden death in this cohort remains unexplained, prompting questions about underlying mechanisms. Here, we present a patient with anti-IgLON5 disease who additionally suffered from transthyretin (ATTR)-amyloidosis and died after cardiac arrest. Furthermore, the literature search of causes of death in the context of anti-IgLON5 disease was conducted and analyzed.

## 2. Materials and Methods

### 2.1. Case Description

We collected information from clinic consultations and discharge letters on one patient with anti-IgLON5 disease who was diagnosed and treated at the Department of Neurology at University Hospital Graz, Austria. Longitudinal serological data of the patient were previously published [7]. The case description follows the CARE case reports guidelines and includes relevant clinical data, diagnostic results, applied therapies, and the course of anti-IgLON5 disease in a patient. Ethical guidelines and data protection were strictly followed. Approval was obtained from the institutional review board of the Medical University of Graz (36-007 ex 23/24).

### 2.2. Systematic Literature Research

The systematic literature review focuses on causes of death in anti-IgLON5 disease. The literature was searched via PUBMED, and the following search terms were used: (iglon5) OR (anti-iglon5) OR (anti iglon5) OR (anti-iglon5-disease) OR (anti-iglon5 disease) OR (anti iglon5 disease) OR (iglon5-disease) OR (iglon5 disease).

**Figure 1:**
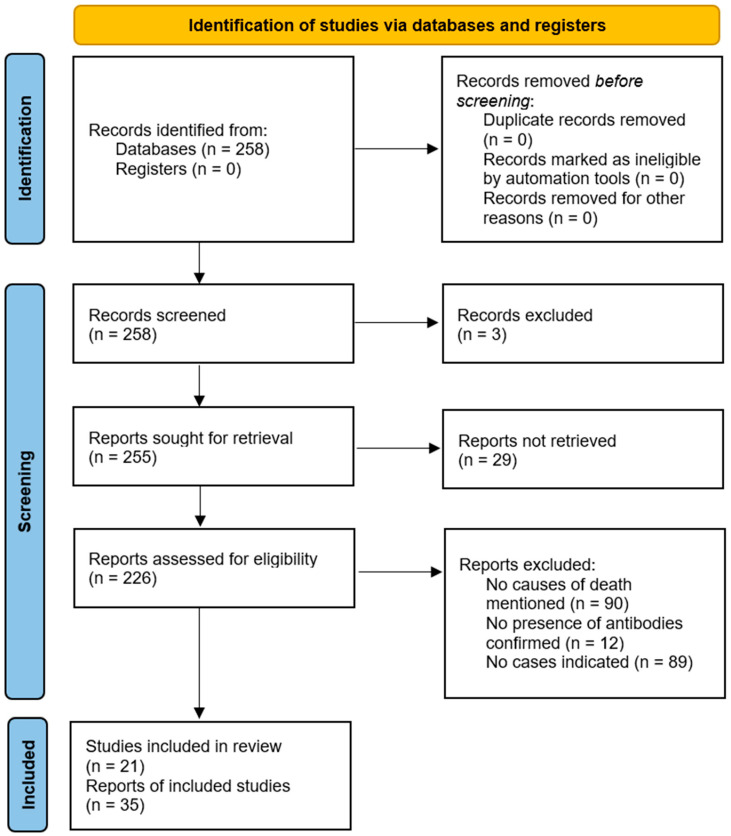
Search strategy of literature review based on the PRISMA 2020 flow diagram for systematic reviews adopted from Page et al. [8].

The literature search was conducted according to the Preferred Reporting Items for Systematic Reviews and Meta-Analyses (PRISMA) guidelines [8]. All publications available up to 1 December 2024, were reviewed, resulting in a total of 258 articles (Figure 1). Only articles in German or English, or those that contained at least English abstracts and provided sufficient information, were included. In order to check the relevance of the publications, both abstracts and full articles were screened and searched using terms such as “IgLON5”, “death”, or “died”. Cases in which there was no evidence of anti-IgLON5 antibodies were excluded. Additionally, articles were excluded if the causes of death were not stated, or no specific cases were reported. References within the articles were also screened for relevance using titles, abstracts, and search terms. We found 61 published cases in a total of 21 publications which reported the causes of death and met the selection criteria. Table 1 summarizes the identified cases and their causes of death. The search was performed by T.H.

## 3. Case Report

A 69-year-old man, diagnosed with Parkinson’s disease four years prior, presented to the University Hospital in Graz, Austria in January 2013 with a chief complaint of impaired fine motor skills.

He had first noticed a lack of motivation several years before, followed by problems when turning, a change in posture, and difficulties when buttoning shirts or performing fine motor tasks. This had led to a diagnosis of Parkinson’s disease in 2009. The patient also reported worsening of his handwriting and a right-sided postural tremor, yet no tremor at rest. Moreover, the patient had noticed subtle difficulties with short-term memory and remembering names. Other symptoms he reported were mild difficulties in word-finding, loss of spontaneity, unsatisfactory fragmented sleep, and fasciculations in the right calf and left shoulder. His previous medical history included depression, lumbar spinal stenosis, arterial hypertension, and mild OSAS. His family history was negative for Parkinson’s disease or any other movement disorders. At the time of presentation, the patient’s medication included levodopa/carbidopa and ropinirole to treat symptoms of suspected Parkinson’s disease. However, there was no clear symptomatic benefit, and discontinuation of the medication remained without subsequent worsening of symptoms.

**Figure 2:**
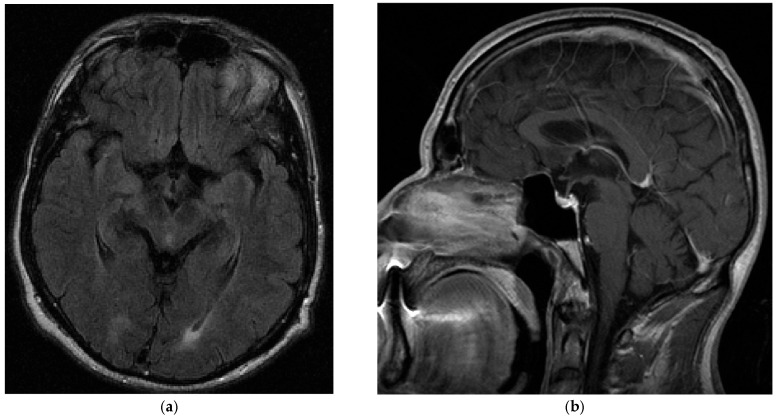
Cerebral MRI at baseline. (**a**) Axial fluid attenuated inversion recovery (FLAIR) sequences showing normal midbrain anatomy. (**b**) Sagittal contrast-enhanced T1-weighted sequences demonstrating normal brain stem anatomy.

Baseline neurological examination revealed a mild bilateral upper limb postural and kinetic tremor; there was no resting tremor. There was slightly increased tone in the right extremities, but no definite bradykinesia. Pallesthesia was mildly reduced in both lower limbs. An MRI of the brain (Figure 2) was reported to be unremarkable, and dopamine transporter single-photon emission computed tomography (SPECT) showed no dopaminergic deficit. The patient scored 29 out of 40 points in the University of Pennsylvania Smell Identification Test, indicating mild hyposmia. Electroneurography indicated incipient sensorimotor axonal polyneuropathy, which was progressive in a subsequent study several months later.

Three and a half years later, in December 2016, the patient first reported restricted mouth opening. He had also noticed impaired horizontal gaze and experienced an increased tendency to fall. Neurological examination at that time revealed an additional supranuclear horizontal gaze palsy and jaw-closing dystonia. Vertical gaze was not significantly impaired, although vertical saccades were slowed. There was also a mild rigidity of the neck and the right upper extremity and mild bradykinesia in the right upper extremity. A slightly unsteady and wide-based gait was noticed. A syndromic diagnosis of “atypical progressive supranuclear palsy (PSP)” was made, although the horizontal gaze palsy was recognized as highly unusual and a red flag against the classic course of PSP.

**Figure 3:**
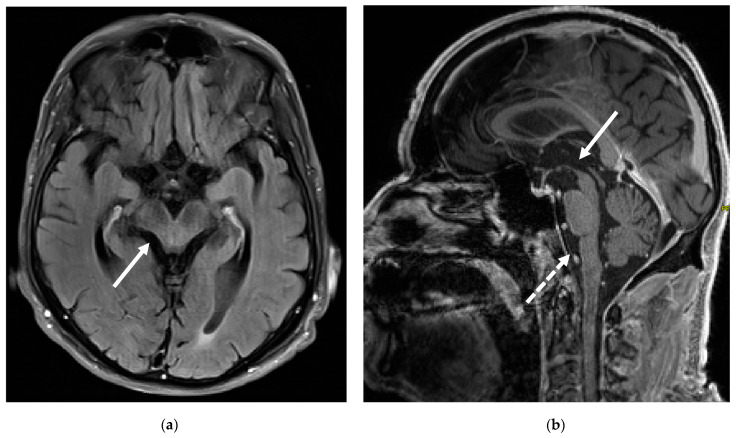
Cerebral MRI at follow-up four years after baseline MRI. (**a**) Axial FLAIR sequences showing midbrain atrophy (white solid arrow). (**b**) Sagittal contrast-enhanced T1-weighted sequences now demonstrating atrophy of the midbrain (white solid arrow) and the medulla oblongata (white dotted arrow).

A clinical follow-up another six months later, in mid-2017, further revealed the development of prominent dysphagia and dysarthria. Jaw-closing dystonia was still present, with a maximum mouth opening of 2 cm. Mild bradykinesia was present in the right more than the left extremities. A cerebral MRI four years after the initial study in 2013 now revealed atrophy of the brain stem, particularly the midbrain (Figure 3). Additionally, a repeated dopamine transporter SPECT now indicated a moderately reduced tracer uptake in the posterior basal ganglia on the right, with a somewhat inhomogeneous tracer uptake in the left basal ganglia, confirming dopaminergic degeneration. Fluoro-deoxy-glucose positron emission tomography did not reveal any alterations in cerebral metabolism. Genetic testing for spinocerebellar ataxias and Niemann–Pick disease type C type 1/2 came back negative.

**Figure 4:**
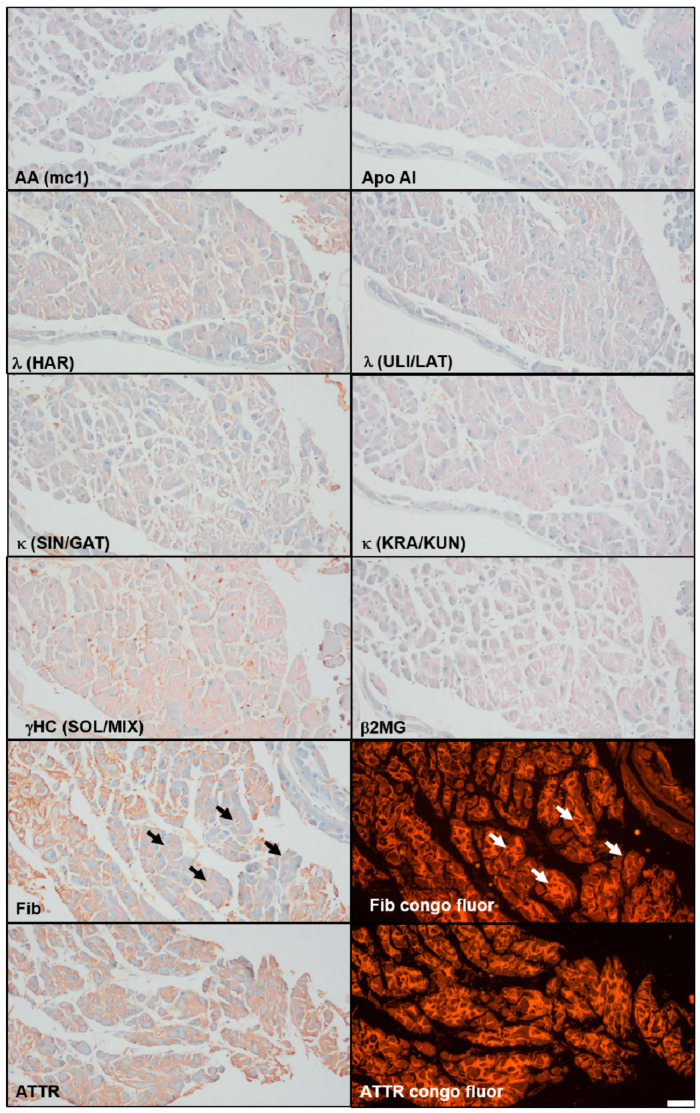
Amyloid typing of the cardiac biopsy using immunohistochemistry (amYmed antibodies) on sections pre-stained with congored [9]: consistent positive staining can only be observed using the antibody specific for ATTR (bottom of image, left ATTR, right congored fluorescence on identical section showing bright positivity), whereas inconsistent staining (arrows) is shown using the antibody against fibrinogen. AA = serum amyloid A, Apo AI = apoprotein AI, λ = Lambda light chain, k = Kappa light chain, gHC = gamma heavy chain, ß2 MG = beta 2 microglobulin, Fib = fibrinogen, and ATTR= transthyretin amyloid. Abbreviations in brackets depict antibody clone used. Bar represents 50 μm; all images taken at original magnification ×20.

In early 2018, the patient had to be hospitalized for two weeks due to worsening dysphagia and an increasing shortness of breath. A cardiac ultrasound indicated an impaired left ventricular function and restrictive cardiomyopathy, and, subsequently, a coronary angiography and endomyocardial biopsy were performed. A suspicion of cardiac amyloidosis was confirmed histologically, and an immunohistochemistry revealed ATTR-amyloidosis (Figure 4), while genetic testing of the TTR gene excluded hereditary ATTR amyloidosis. Treatment with Tafamidis was initiated. An MRI of the orbits did not indicate ocular amyloidosis.

Additionally, the patient was diagnosed with severe glottic stenosis in March 2019, which supposedly had led to increased dyspnea and nocturnal pauses in breathing; an outpatient polygraphy had revealed an apnea–hypopnea index of 62; and the patient consequently received a tracheotomy. At the same time, a third-degree atrioventricular block led to the implantation of a pacemaker system, and coronary artery stenoses were treated with drug eluting stents.

In October 2019, serum was tested for the first time for the presence of anti-IgLON5 antibodies with a tissue-based assay (primary dilution 1:200) and live cell-based assay (primary dilution 1:40), as previously described [1,7]. A positive result was obtained with a titer of 1:12,800. HLA-DRB1*01:01 and HLA-DQB1*05:01 alleles were present in the patient’s HLA typing. Intravenous immunoglobulins (0.4 g per kilogram of body weight) were administered for 5 days without apparent beneficial effects. Botulinum toxin treatment of the jaw-closing dystonia did not relieve symptoms.

In January 2020, the patient reported further progression of dysphagia, jaw-closing dystonia, and bradykinesia and was subsequently hospitalized. There was also an increase in the frequency of falls, daytime tiredness, and depressive mood. A follow-up serologic test of the anti-IgLON5 titer was still positive at 1:1600 yet lower than before. The antibody titer in the CSF was 1:16 (primary dilution 1:2). The patient showed a robust intrathecal anti-IgLON5 IgG4 synthesis, based on corresponding data for CSF/serum IgG and the albumin ratio and calculation of z-scores for anti-IgLON5 IgG4 and total IgG, as described previously [7]. A second cycle of intravenous immunoglobulins was administered, and a percutaneous endoscopic gastrotomy tube had to be placed because of progressive dysphagia. Due to presumed aspiration, the patient went into cardiac arrest during hospitalization but was successfully resuscitated and finally recovered to his previous clinical state. In March 2020, the patient was released into palliative care and received long-term oxygen therapy and invasive out-of-hospital ventilation. Two years later, the patient suffered a cardiac arrest at home and was resuscitated with the return of spontaneous circulation. However, he remained comatose and died a few weeks later.

## 4. Results of the Literature Review

**Figure 5:**
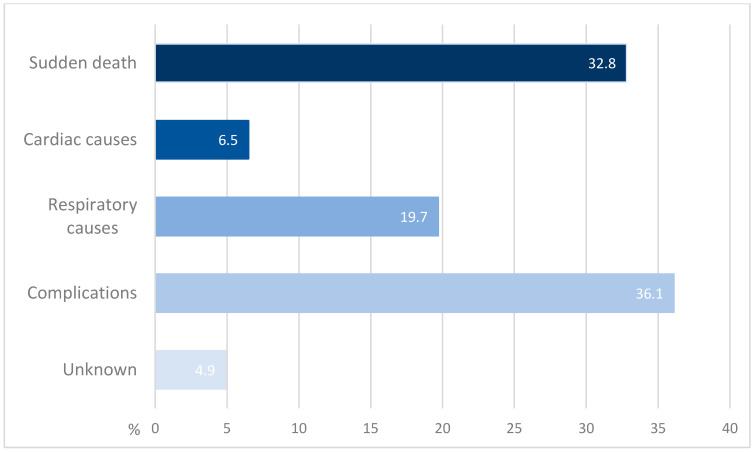
Causes of death in published cases of anti-IgLON5 disease (if stated).

A total of 61 cases with documented causes of death were analyzed. Causes of death are categorized as sudden death, cardiac causes, respiratory causes, complications of the disease, and unknown causes. The percentage distribution of reported causes of deaths is summarized in Figure 5. The most prevalent cause was death due to complications of the disease, with a percentage of 36.1% of all the described causes of death [2,6,10,11,12,13,14,15,16]. This category includes aspiration [6], aspiration pneumonia/pneumonia [2,11,13,14,16], complications following a traumatic fall [6,10], pulmonary embolism [6], sepsis [10], cerebral hemorrhage [12], urinary infection and sepsis [16], COVID-19 infection [15] and progression of a hypernephroma, although no additional details clarify whether the latter is related to anti-IgLON5 disease [2]. A total of 14 out of 22 of the complication-related cases are attributed to aspiration pneumonia/pneumonia [2,11,13,14,16], representing 23% of all documented causes of death.

**Table 1:** Causes of death in published cases of anti-IgLON5 disease.

Publication	Causes of Death	Notes
Gaig et al. [2]	Sudden death (*n* = 6) Aspiration pneumonia (*n* = 6) Hypernephroma progression (*n* = 1)	22 patients in total, 13 died (59%). Of the patients who died because of sudden death, two died during wakefulness, two while sleeping, and for two, the time was unknown. Heart complications in 4 patients.
Erro et al. [17]	Sudden death (*n* = 1)	
Sista et al. [18]	Sudden death (*n* = 1) Cardiac arrest (*n* = 1)	Case reports of four patients, two died.
Grüter et al. [6]	Central hypoventilation (*n* = 4) Aspiration (*n* = 1) Complications after traumatic fall (*n* = 1) Cardiac arrhythmia (*n* = 1) Pulmonary embolism (*n* = 1) Unclear reason (*n* = 2)	52 patients in total, 10 died.
Sabater et al. [1]	Sudden death (*n* = 5)	Study with 8 patients. Two of the five patients who died because of sudden death were awake, two were asleep. For the other sudden death, there is no more precise information available. For the other three patients there is no further information either; one died in the intensive care unit, but no reason is given.
Bahtz et al. [19]	Sudden death (*n* = 1)	
Högl et al. [11]	Aspiration pneumonia (*n* = 1)	
Schröder et al. [20]	Cardiac infarction (*n* = 1)	Reference was made to a recent study, but no exact source was given. Comorbidities were type 2 diabetes and arterial hypertension.
Wenninger et al. [21]	Sudden death (*n* = 1)	During sleep.
Honorat et al. [4]	Respiratory failure (*n* = 2)	The paper reports 4 deaths but only mentions the causes of two patients.
Berger-Sieczkowski et al. [10]	Subdural hemorrhage after a fall (*n* = 1) Pneumonia/sepsis (*n* = 1) Respiratory failure (*n* = 1)	The paper also reports other cases which are already mentioned above.
Asioli et al. [22]	Sudden death (*n* = 1)	During sleep.
Della Marca et al. [23]	Sudden death (*n* = 1)	During sleep.
Chen et al. [24]	Sudden death (*n* = 1)	
Liu et al. [12]	Cerebral hemorrhage (*n* = 1)	
Montojo et al. [13]	Aspiration pneumonia (*n* = 1)	
Klein da Costa et al. [14]	Pneumonia (*n* = 1)	Septic shock secondary to pneumonia.
Li et al. [25]	Sudden death (*n* = 1)	
Gelpi E. et al. [16]	Pneumonia (*n* = 4) Respiratory failure (*n* = 4) Respiratory insufficiency (*n* = 1) Unknown (*n* = 1) Urinary infection and sepsis (*n* = 1)	22 cases in total, 10 cases were already published. From the 12 remaining cases, one was excluded because there was no antibody testing. One patient with pneumonia developed sepsis, one patient with pneumonia developed status epilepticus. The patient with the unknown cause was found dead at home.
Postuma R. et al. [15]	COVID-19 infection (*n* = 1) Sudden death (*n* = 1)	The patient with sudden death required intubation, became comatose, and died.
Cagnin et al. [26]	Bradycardia (*n* = 1)	The patient had recurrent hypoxic events during the disease.

With 32.8%, the second most common cause is sudden death [1,2,15,17,18,19,21,22,23,24,25]. Within this group, a further distinction was made between sudden death during wakefulness, during sleep, and at an unknown time. In this further subdivision, sudden death at an unknown time occurred most frequently (45%) [1,2,15,17,18,19,24,25], followed by sudden death during sleep with 35% [1,2,21,22,23]. Sudden death during wakefulness occurred in 20% of cases [1,2].

Next, respiratory causes can be identified as contributing factors to mortality, accounting for 19.7% of cases [4,6,10,16]. Causes in this subgroup are respiratory failure [4,10,16] and central hypoventilation [6], which occurred seven and four times, as well as respiratory insufficiency [16], which occurred once, respectively.

Cardiac causes are reported in 6.5% of cases [6,18,20,26]. Four cases are described involving a distinct cardiac event: an attack with extreme bradycardia [26], cardiac arrest [18], arrhythmia [6], and infarction [20]. The patient who died of cardiac infarction had comorbid type 2 diabetes and arterial hypertension [20].

Lastly, unknown causes were reported at 4.9% [6,16]. Additional insights emerge from various publications: for instance, in the study by Gaig et al. involving 22 patients, 13 deaths were recorded, resulting in a fatality rate of 59%. The observation period ranges from 0 to 48 months, with an average of 11.8 months. Six of the thirteen patients experienced sudden death, and an additional four patients showed signs of cardiac complications, although the specific individuals affected were not clearly specified. The median age at diagnosis was 64 years, ranging from 46 to 83 years; however, individual disease duration until death was not specified [2]. In another study involving 52 patients, 10 died within 3 years after disease onset, indicating a fatality rate of 19.2%, which is notably lower than in the previously described study [6].

Individual disease duration until death (Table 2) was reported in 27 cases [1,10,11,16,17,18,19,21,22,23,26] and ranged from 2 months [1] to 180 months [16]. The median disease duration until death tended to be shortest in cases of sudden death (10 cases, 24 months), followed by cases of death due to complications (8 cases, 34.5 months), cardiac causes (2 cases, 49.5 months) and respiratory causes (6 cases, 84 months).

## 5. Discussion

### 5.1. Case Report

Our patient’s epidemiological profile aligns with the previous literature. At 69 years old, he falls within the range of 46 to 83 years for the age of diagnosis [2]. However, the time from motor symptom onset (2009) to diagnosis (2019)—approximately 10 years—was significantly longer than the reported average of 33.3 ± 37.5 months [6]. Since non-motor features like lack of motivation, retrospectively possibly attributable to anti-IgLON5 disease, had already been present several years before the initial diagnosis of Parkinson’s disease, an even longer duration between symptom onset and final diagnosis could be assumed.

A few months after the initial presentation to our clinic, sensorimotor axonal polyneuropathy was detected, and five years later, cardiac amyloidosis of the ATTR-type was diagnosed. ATTR amyloidosis, known to cause dizziness, syncope, restrictive cardiomyopathy, and progressive terminal heart failure, as well as axonal sensorimotor polyneuropathy [27], may in fact also account for the patient’s peripheral neuropathy. Other cases with anti-IgLON5 disease and sensorimotor polyneuropathy were described, though the presence or absence of cardiac amyloidosis in these cases is unclear [28].

Oromandibular dystonia and restricted mouth opening was also present in our patient and consistent with reported cases [22], as well as horizontal gaze palsy [3], as seen in our patient.

During the course of the disease, the patient reported frequent falls. This could be attributed to a high level of daytime sleepiness caused by unsatisfactory sleep and OSAS as well as gait instability. Irresistible sleep attacks are also characteristic of anti-IgLON5 disease and occur in 30–59% of patients [6].

The brain MRI of our patient at initial presentation to our clinic was unremarkable, aligning with most reports. The follow-up MRI several years later, however, did reveal brain stem atrophy, suggesting that visible structural changes may emerge at more advanced stages. Abnormal MRI findings were found in only 19% of cases in the literature, which mainly consist of atrophy of the brain stem and cerebellum; post-mortem studies found tau deposits in addition to these regions in the hypothalamus and hippocampus [3]. Erro et al. reported a patient with classic symptoms and typical HLA and serological findings, who, however, did not exhibit tauopathy on the post-mortem work-up. This could be an indication of an immune-mediated mechanism that precedes the presence of p-tau deposits, with tauopathy representing a secondary event [17]. Sleep disorders can be explained by p-tau deposits in the hypothalamus and hippocampus. Movement disorders and gait instability, as well as oculomotor impairments, can also occur due to brain stem involvement [3]. However, the early onset of these symptoms in the absence of MRI abnormalities is most likely attributable to neuronal dysfunction mediated by antibodies.

The main diagnostic criterion for anti-IgLON5 disease is the detection of antibodies in serum and CSF. In most cases previously reported, both serum and CSF were analyzed. Initially, only serum was tested in our patient, yielding a titer of 1:12,800. This lies within the published ranges, though it is higher than the median 1:640 [6], with titers of up to 1:20,480 reported [6]. Following immunoglobulin therapy, the titer fell to 1:1600 without clinical improvement, implying that irreversible neuronal damage may have occurred before treatment initiation. The CSF analysis performed later also revealed IgLON5 antibodies at a titer of 1:16, consistent with the reported ranges (1:16–1:256) [6]. To prevent irreversible neuronal changes such as tau accumulation, early diagnosis and treatment with immunotherapy are deemed essential [2].

HLA typing also provides additional insights. HLA-DRB1*10:01 and HLA-DQB1*05:01 are deemed characteristic for the disease [2]; our patient carried HLA-DQB1*05:01 and HLA-DRB1*01:01, which is one of the two typical HLA-alleles [6] previously found in anti-IgLON5 patients [29]. Typical HLA findings correlate with symptoms such as REM sleep disorder, non-REM parasomnia, daily sleep attacks, and sleep breathing disorder, less commonly found in HLA-negative patients [6]. In a study by Yogeshwar et al., however, no association was found between HLA-DRB1*10:01 and increased sleep or bulbar symptoms. A retrospective analysis of sleep and HLA data revealed that HLA-DQB1*05:01 occurs particularly frequently in various forms of non-REM parasomnias. This suggests that the classification of certain sleep disorders is more likely related to the individual HLA-DQB1*05 allele and not necessarily to specific combinations of HLA haplotypes [30].

In previous studies, many patients with anti-IgLON5 disease are treated with monthly cycles of intravenous immunoglobulins or steroids [2]. Although initial improvement of symptoms has been reported, some patients show no or limited response [4], mirroring our patient’s experience. Treatments with plasma exchange, rituximab, and cyclophosphamide were also reported [31]. Despite a lack of clinical effect in our patient, the serum titer decreased from 1:12,800 to 1:1600, resembling previous reports where titers dropped or even became negative [32].

Our patient also suffered from cardiac complications. Due to aspiration—a common complication [3]—the patient went into cardiac arrest but was successfully resuscitated. A subsequent cardiac arrest, however, left him comatose, and he died a few weeks later. Cardiac arrests and other cardiac complications are repeatedly reported in patients with anti-IgLON5 disease, and Gaig and colleagues have suggested a link between cardiac complications and sudden death in these patients [2]. In our case, coexisting ATTR amyloidosis may have exacerbated these complications, potentially reinforcing the suspected association between anti-IgLON5 disease, cardiac manifestations, and mortality.

### 5.2. Causes of Death

There is a wide spectrum of causes of death in anti-IgLON5 disease. Most of them can be attributed to specific symptoms or their exacerbation. This does not hold true for the frequent cause sudden death, which occurs in 32.8% of cases. Sudden death is documented with a similar frequency during wakefulness (20%), sleep (35%), and at an unknown time (45%), offering no clear pathophysiological insights. Due to limited data—particularly regarding deaths at unknown times—it seems plausible that some of these cases could be reclassified if more precise information was available. Even then, no definitive conclusions could be drawn regarding the pathomechanism behind these sudden deaths. However, Gaig et al. reported both sudden deaths (two during wakefulness, two during sleep, and two at unknown times) and cardiac complications in four patients (ventricular tachycardia, bradycardia requiring a pacemaker, and Takotsubo cardiomyopathy) in their cohort, suggesting a possible link [2]. Our patient, who had ATTR amyloidosis and experienced sudden cardiac death, further supports this suspicion. It is conceivable that some cases of sudden death may actually represent sudden cardiac death due to overlooked cardiac conditions, emphasizing the need for greater attention to cardiac comorbidities and potentially improving outcomes through earlier intervention.

The most common cause of death (36.1%) is death due to disease-related complications. The main cause in this category is pneumonia, which was mainly associated with aspiration [2,11,13]. Aspiration pneumonia/pneumonia accounts for 23% of all cases and could warrant its own subcategory, since its frequency might inflate the overall complication rate. This is unsurprising, given that aspiration and pneumonia are common throughout the disease course [4]. Trauma-related deaths, such as from falls, can be explained by prevalent gait instability [6].

Respiratory causes account for 19.7% of deaths, and patients often exhibit respiratory compromise requiring intensive care treatment or tracheostomy during the disease course [2,33]. Central hypoventilation and respiratory failure have been described as direct respiratory causes of death [2].

Death from definite cardiac causes is rare (*n* = 4) and heterogeneous (bradycardia, cardiac arrest, and arrhythmia, infarction), further complicating pattern recognition. Still, given the prevalence of cardiac issues in anti-IgLON5 disease [2] and the possible connection between these complications and sudden death, further research is warranted. Some proportion of sudden deaths might indeed be cardiac in origin, as suggested by Gaig et al. [2] and further supported by our findings.

Disease duration until death varied greatly between cases (Table 2). Cases of sudden death tended to have the shortest disease duration until death, while respiratory causes were characterized by the longest duration. However, disease duration until death was only reported in 27 of 61 cases [1,10,11,16,17,18,19,21,22,23,26]. Our case retrospectively first developed symptoms in 2009 and died in 2022, yielding an approximately 13 years disease duration until death, a figure in the upper spectrum of previously reported cases.

Further investigation of the relationship between cardiac complications and sudden death in anti-IgLON5 disease may lead to optimized diagnostic and therapeutic strategies in the future and ultimately improve the quality of life for affected patients.

## Figures and Tables

**Table 2:** Disease duration until death in individual cases (if reported), sorted by cause of death.

Cause of Death	Age at Disease Onset	Disease Duration Until Death
Sudden death [1]	65	2 months
Sudden death [1]	76	6 months
Sudden death [19]	71	7 months
Sudden death [21]	58	24 months
Sudden death [22]	68	24 months
Sudden death [1]	69	24 months
Sudden death [17]	71	24 months
Sudden death [1]	53	72 months
Sudden death [23]	55	120 months
Sudden death [1]	59	144 months
Pneumonia/sepsis [16]	81	6 months
Pneumonia/sepsis [10]	82	6 months
Pneumonia [16]	77	9 months
Urinary infection/sepsis [16]	85	9 months
Infection [16]	66	60 months
Pneumonia [16]	61	108 months
Aspiration pneumonia [11]	54	144 months
Pneumonia [16]	62	156 months
Respiratory insufficiency [16]	81	11 months
Respiratory failure [16]	69	48 months
Respiratory failure [16]	70	72 months
Respiratory failure [16]	66	96 months
Respiratory failure [10]	76	108 months
Respiratory failure [16]	50	180 months
Bradycardia [26]	69	15 months
Cardiac arrest [18]	53	84 months
Unknown [16]	75	6 months

## Data Availability

The data presented in this study are available on request from the corresponding author due to privacy reasons.

## Abbreviations

The following abbreviations are used in this manuscript:

ATTR	Amyloid transthyretin
CSF	Cerebrospinal fluid
FLAIR	Fluid attenuated inversion recovery
HLA	Human leukocyte antigen
MRI	Magnetic resonance imaging
OSAS	Obstructive sleep apnea syndrome
PRISMA	Preferred Reporting Items for Systematic Reviews and Meta-Analyses
PSP	Progressive supranuclear palsy
REM	Rapid-eye-movement
SPECT	Single-photon emission computed tomography
